# Structural basis for the interaction between human coronavirus HKU1 spike receptor binding domain and its receptor TMPRSS2

**DOI:** 10.1038/s41421-024-00717-5

**Published:** 2024-08-08

**Authors:** Xiaopan Gao, Kaixiang Zhu, Lin Wang, Kun Shang, Lei Hua, Bo Qin, Hongtao Zhu, Wei Ding, Sheng Cui

**Affiliations:** 1 NHC Key Laboratory of Systems Biology of Pathogens, National Institute of Pathogen Biology, Chinese Academy of Medical Sciences and Peking Union Medical College, Beijing, China; 2 Key Laboratory of Pathogen Infection Prevention and Control (Ministry of Education), National Institute of Pathogen Biology, Chinese Academy of Medical Sciences & Peking Union Medical College, Beijing, China; 3 State Key Laboratory of Respiratory Health and Multimorbidity, National Institute of Pathogen Biology, Chinese Academy of Medical Sciences & Peking Union Medical College, Beijing, China; 4 Beijing National Laboratory for Condensed Matter Physics, Institute of Physics, Chinese Academy of Sciences, Beijing, China; 5 Songshan Lake Materials Laboratory, Dongguan, Guangdong China; 6 Medical School, Yan’an University, Yan’an, Shaanxi China; 7 University of Chinese Academy of Sciences, Beijing, China

**Keywords:** Cryoelectron microscopy, Cell signalling

Dear Editor,

HKU1 coronavirus (CoV) infection can be severe in children, elderly, or immunocompromised patients^1^. As a member of the *Embecovirus*, HKU1 uses a primary glycan-based receptor (9-*O*-acetylated sialosides) and a secondary proteinaceous receptor for host cell targeting and membrane fusion^2,3^. One unique feature of HKU1 spike is that the C-terminal domain (CTD) of S1 (also known as receptor-binding domain, RBD) is exceptionally larger than other CoV RBDs, and the receptor-binding motif (RBM) of HKU1 adopts a unique fold^4^. A recent cryogenic electron microscopy (Cryo-EM) study revealed that binding of a 9-*O*-acetylated α2,8-linked sialic acid to the HUK1 spike S1-NTD leads to an allosteric switching of the adjacent RBD to “up” conformation, thereby allowing secondary receptor binding^3^. Proteinaceous receptor of HKU1 was identified as transmembrane serine protease 2 (TMPRSS2) and HKU1 RBD binds TMPRSS2 with nanomolar affinity^2^. Nevertheless, detailed mapping of the interaction between HKU1 spike and TMPRSS2 remains unavailable.

To identify key regions mediating HKU1 spike–TMPRSS2 interaction, we purified HKU1 RBD and TMPRSS2 ectodomain and assembled the binary complex (Fig. 1a; Supplementary Fig. S1a, b). Both HKU1-A (1A) and HKU1-B (1B) interact with TMPRSS2; we therefore assembled 1A-RBD–TMPRSS2 and 1B-RBD–TMPRSS2 complexes, respectively. Using bio-layer interferometry (BLI), we determined the equilibrium dissociation constant (*K*_D_) for RBD–TMPRSS2 interaction (Fig. 1b, c). The affinity of 1A-RBD–TMPRSS2 (*K*_D_ = 323 nM) was ~2-fold lower than that of 1B-RBD–TMPRSS2 (*K*_D_ = 170 nM), which is consistent with the *K*_D_ values reported previously^2^.

**Fig. 1:**
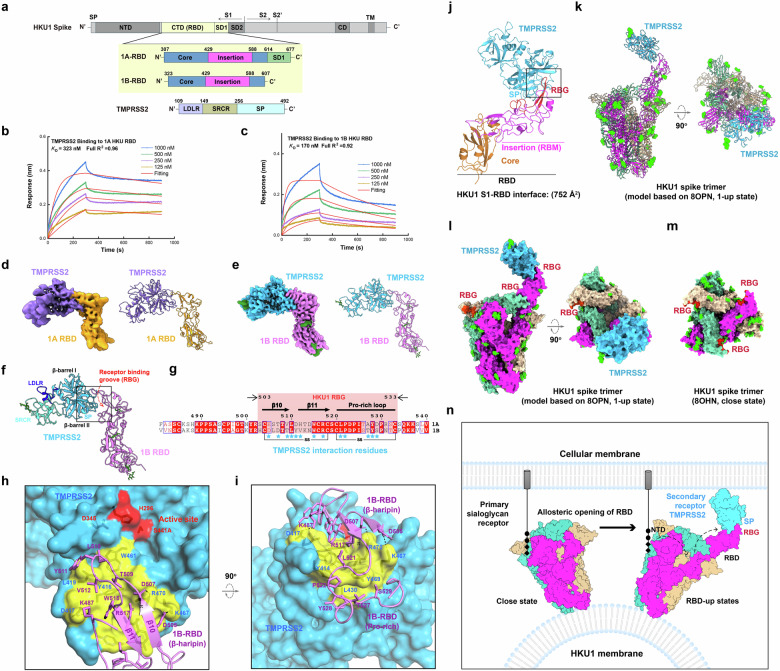
Cryo-EM structures of HKU1 spike S1-RBD in complex with TMPRSS2. **a** Schematic diagrams of HKU1 spike S1-RBD and TMPRSS2 constructs. **b**, **c** BLI titrations of 1A RBD–TMPRSS2 (**b**) and 1B RBD–TMPRSS2 (**c**); the *K*_D_ values of binding are indicated. Data fitting is shown with red curves. **d** Cryo-EM structure of HKU1 1A RBD (orange) in complex with TMPRSS2 (purple); left, cryo-EM densities, right, ribbon models. **e** Cryo-EM structure of HKU1 1B RBD (magenta) in complex with TMPRSS2 (blue); left, cryo-EM densities, right, ribbon models. Sugars are colored green. **f** 1B RBD and TMPRSS2 interaction shown with ribbon models. HKU1 receptor binding groove (RBG) is indicated. **g** Sequence alignment of 1A-RBD and 1B-RBD illustrating 13 residues (marked by blue asterisks) of RBG important for TMPRSS2 binding. Two disulfide bridges stabilizing RBG are indicated. **h**, **i** Detailed interaction between HKU1 1B RBD and TMPRSS2. TMPRSS2 is shown with molecular surface (blue), the proteinase active site is colored red, and RBD binding site is colored yellow. HKU1 1B RBD is shown with ribbon model (magenta). Key interaction residues are shown with stick model. Magnified view focusing 1B-RBD β-hairpin–TMPRSS2 interaction (**h**) and 1B-RBD Proline-rich–TMPRSS2 interaction (**i**) are shown. **j** HKU1 RBD–TMPRSS2 complex. RBD core domain is colored orange, RBG is colored red, and the insertion domain (RBM) is colored magenta. The black box indicates the RBD–TMPRSS2 interface. **k**, **l** TMPRSS2 (blue) is modeled to an HKU1 spike trimer with one RBD in up-state. Ribbon model (**k**) and molecular surface (**l**) are shown. Sugars are shown with green spheres; the RBGs are colored red. **m** Top view of an HKU1 spike trimer in close-state. RBGs are colored red. **n** A working model of HKU1 spike protein-mediated receptor binding.

We determined the cryo-EM structures of 1A-RBD–TMPRSS2 and 1B-RBD–TMPRSS2 complexes (Supplementary Figs. S2, S3 and Table S1). Reconstruction of 1A-RBD–TMPRSS2 particles yielded poor EM densities, whereas reconstruction of 1B-RBD–TMPRSS2 particles yielded clearer densities (Fig. 1d, e; Supplementary Fig. S4), despite the resolution of 1A-RBD–TMPRSS2 map estimated by gold-standard Fourier shell (3.34 Å) is similar to that for 1B-RBD–TMPRSS2 map (3.95 Å). As shown in Fig. 1d, e, the map quality for 1A-RBD–TMPRSS2 is obviously lower than that for 1B-RBD–TMPRSS2. Given that high-resolution crystal structures of HKU1A RBD and TMPRSS2 were separately determined by our group and other groups^4,5^, we fitted the HKU1A RBD and TMPRSS2 atomic model into our HKU1A RBD–TMPRSS2 EM density map by rigid body fitting. The fitting was unambiguous and the models fitted the EM density well. The difference between 1A-RBD–TMPRSS2 and 1B-RBD–TMPRSS2 maps may attribute to higher binding affinity of 1B RBD for TMPRSS2 (Fig. 1b, c) that enhances stability of the complex. Therefore, we mainly used 1B-RBD–TMPRSS2 structure for subsequent structural analysis.

To investigate whether the binding mode of TMPRSS2 with 1A-RBD and 1B-RBD, and the regions involved in the TMPRSS2–RBD interaction are similar, we superimposed two structures (Supplementary Fig. S5a), giving an overall RMSD (Cα atoms) value of 1.3 Å. Further, we compared the structures of TMPRSS2 regions (residues 405–421, 427–436, 461–472) directly involved in binding RBD, which gave an RMSD value of 0.7 Å; we also compared the structures of RBD regions (residues 485–491, 502–535) directly involved in binding TMRPSS2, which gave an RMSD value of 0.9 Å (Supplementary Fig. S5b). All RMSD values of the structural comparison are listed in Supplementary Table S2.

Although our map for HKU1A RBD–TMPRSS2 was adequately resolved and facilitated unambiguous rigid body fitting of HKU1A RBD and TMPRSS2 models, we were not confident for its accuracy. Thus, we superimposed our TMPRSS2-1A RBD model with a recently released TMPRSS2-1A RBD structure with higher resolution (Protein Data Bank (PDB) 8VGT, 2.9 Å) to ensure the quality of our model. The comparison gave an RMSD of 0.8 Å, indicating that two models are highly similar (Supplementary Fig. S5c). Further, we compared the structures of interaction regions in 1 A and 1B RBD that directly contact TMPRSS2, which gave an RMSD of 0.9 Å, confirming that this region adopts a similar fold despite amino acid variances between HKU1 1 A and 1B. The RMSD values for comparison of various TMPRSS2 and HKU1 RBD structures are listed in Supplementary Table S2.

We used TMPRSS2–1B RBD structure to analyze their interaction in detail. The TMPRSS2–RBD interaction occurred between a groove formed on the HKU1 RBD apex and a hydrophobic surface near the active site in the C-terminal trypsin-like serine peptidase (SP) domain of TMPRSS2 (Fig. 1f). We denote this groove: receptor-binding groove (RBG) hereafter (Fig. 1f; Supplementary Fig. S5d). RBG is an arch-shaped module comprising an antiparallel β-hairpin (β10–β11, previously referred to as a horn^4^) and a proline-rich loop (Supplementary Fig. S5d); two disulfide bridges C504–C518 and C520–C533 stabilize the fold of this module (Fig. 1g). We superimposed 1A RBD structure (PDB 5GNB) with TMPRSS2 in complex with 1A RBD and 1B RBD structures (Supplementary Fig. S5d), and the RMSD values are between 0.7 and 0.9 Å (Supplementary Table S2), revealing that HKU1 RBD undergoes negligible conformational change upon receptor binding. We also compared free-state TMPRSS2 structure (PDB 7MEQ) with the structure of TMPRSS2 bound by 1B RBD, showing that the overall structure of TMPRSS2 was largely unaffected, expect for some minor shifts of loops at the interaction interface (Supplementary Fig. S5e). Collectively, our structural investigation demonstrates that the TMPRSS2–RBD binding primarily involves hydrophobic packing and shape complementarity. No major conformational changes were observed.

The TMPRSS2–RBD interactions involve central hydrophobic packings flanked by several salt bridges (Fig. 1h, i). We identified 14 HKU1 spike residues and 8 TMPRSS2 residues mediating the interactions, rendering an interfacial area of 752 Å^2^. In particular, residues T509, L510, Y511, V512 and W515 on the β-hairpin of RBG are packed against a hydrophobic patch on TMPRSS2 formed by residues Y416, L419 and W461 (Fig. 1h). A π-stacking interaction is formed between TMRPSS2 R470 and 1B RBD R517 (Fig. 1h, i). Residues L521, P522, T527, Y528 and S529 from the proline-rich loop of RBG interact with a hydrophobic protrusion formed by L430, Y414 and Y469 of TMPRSS2 (Fig. 1i). Additionally, the salt bridges between HKU1 spike D507 and TMPRSS2 R470, and between HKU1 spike D505 and TMPRSS2 K467 further stabilize the TMPRSS2–RBD interaction (Fig. 1h, i). Lastly, HKU1 spike K487 forms an additional salt bridge with TMPRSS2 D417 (Fig. 1i), and this is the only non-RBG residue contributing to the interaction.

RBG covers a region of 31 amino acids (residues 503–533) in HKU1 RBD (A and B genotypes), on which 13 interaction residues constitute a concave surface for TMPRSS2 binding (Fig. 1g). Six of those are invariant between HKU1 genotypes, including L510, W515, R517, L521, P522 and Y528 (Fig. 1g–i), probably underlying the common receptor binding mode for two HKU1 genotypes. Our structural investigation matches well with the previous functional analyses that the suggested residues 505, 515, 517–521 and 528 of HKU1 spike are important for receptor binding^4^. Variation in RBG sequences could result in minor changes in receptor binding affinity, e.g., residue D507 of 1B RBD is replaced by T507 in 1A RBD; this variation could disrupt a salt bridge between RBD and TMPRSS2, which may explain the decrease in receptor binding affinity (Fig. 1b).

To establish a detailed comparison between 1A and 1B RBD in receptor binding, we superimposed our 1B RBD structure bound by TMPRSS2 with the recently released 1A RBD structure bound by TMPRSS2 (PDB 8VGT, Supplementary Fig. S5c). Except four residues on apex of β-hairpin (residues 509–512) which have minor shifts, all other interaction residues are nearly superimposable (Supplementary Fig. S5c). Thus, the difference between 1A and 1B RBD in receptor binding affinity most likely stems from variation in the side chains of amino acids. Variations D511Y and H512V between 1A and 1B RBD may alter hydrophobic packing interaction; variations S487K and T507D between 1A and 1B RBD may affect salt bridging contacts, which on the whole results in different receptor binding affinity.

HKU1 RBD occupies a vital region in the TMPRSS2 SP domain near the proteinase active site cleft. This region is involved in three variable, surface-exposed loops on the SP domain (Loop 1–3 on β-barrel II) governing substrate specificity. Therefore, it is possible that binding of HKU1 spike affects TMPRSS2 substrate recognition^5^. We also overlaid the structure of TMPRSS2–RBD with the structure of human serine protease hepsin (a TMPRSS2 homolog) bound to the KQLR peptide (PDB 1Z8G)^6^, which implies that the HKU1 RBD could impede binding to the peptide substrate proteinase active site due to steric hindrance (Supplementary Fig. S6).

During submission of this paper, several papers reporting similar structures have published^7–9^. Crystal structure of HKU1 RBD–TMPRSS2 nanobody A01 complex was determined to 3.5 Å^8^. A01 is a non-competing nanobody for TMRPSS2 that does not affect the RBD–TMPRSS2 interactions^8^. The nanobody was developed by the authors to promote crystallization, providing alternative avenue to structure determination. McCallum et al reported a 2.9 Å cryo-EM structure of the HKU1A RBD–TMPRSS2^7^. Xia et al. determined a set of cryo-EM structures including the structure of HKU1 spike trimer with RBD in “up” position and bound by TMPRSS2^9^. All available structural analyses identified a common binding interface, which is between TMPRSS2 SP domain surface loops (L1–L3) and HKU1 RBD receptor binding groove (also known as RBD pincer plier^8^) at the apex of RBD (Fig. 1f), which suggests that binding of HKU1 spike may block TMPRSS2 catalytic groove.

To identify the determinants for TMPRSS2 binding specificity, we aligned sequences of TMPRSS2 orthologs from various species including mammals and birds (Supplementary Fig. S7a). Our Cryo-EM structures revealed that eight TMPRSS2 residues are important for HKU1 RBD binding, including Y414, Y416, D417, L419, L430, W461, Y469 and R470 (Fig. 1g–i). As shown in Supplementary Fig. S7a, D417 and Y469 are variable among TMPRSS2 orthologs and all other residues are invariant, suggesting that D417 and Y469 are important for HKU1 tropism. This analysis is in line with a recently published paper reporting similar results from pseudovirus-based mutagenesis studies^2^. Further, we aligned sequences of various TMPRSS-related proteins in humans, including TMRPSS2, 3, 4, 5 and 13 (Supplementary Fig. S7b). Except for invariant residue Y416, all other residues important for RBD interaction are variable among TMPRSS-related proteins, suggesting that TMPRSS2 is specific for HKU1 RBD binding.

TMPRSS2 polymorphisms were investigated for susceptibility to SARS-CoV-2 infection^10^. We analyzed several single nucleotide polymorphisms (SNPs) that cause missense mutation in TMPRSS2 proteins from NCBI dbSNP database. None of those SNPs overlaps with the eight key RBD interaction residues identified in our study (Supplementary Table S3). How TMPRSS2 polymorphism affects HKU1 susceptibility requires in-depth investigation in the future.

All CoV RBDs possess a core domain containing a five-stranded antiparallel β-plane and connecting loops. The longest loop connecting the 4th and 5th β-strands of the β-plane folds into RBM defining the receptor binding interface (Fig. 1j; Supplementary Fig. S8a–d). Although the core domain of HKU1 RBD exhibits a similar fold, the loop topologically equivalent to RBM exhibits a unique fold (Fig. 1j; Supplementary Fig. S8). Although HKU1 spike has the largest RBM, only a small portion of it forms the RBG for receptor binding, rendering an interfacial area of only 752 Å^2^, smaller than other CoV RBM–receptor interfacial areas (846–978 Å^2^, Fig. 1j; Supplementary Fig. S8a–d). The rest of the RBM structure may play roles other than receptor binding, which will be interesting to investigate.

To gain an overview of HKU1 receptor binding, we modeled the TMPRSS2 structure onto an HKU1 spike trimer with one RBD in up-state and two RBDs in close-state (PDB 8OPN, Fig. 1k, l) by superimposing our TMPRSS2-RBD structure onto it. It is obvious that TMPRSS2 can interact with HKU1 RBG without steric hindrance when RBD is flipped up. However, in the close-state, spike RBG is not fully buried between adjacent protomers (Fig. 1l, m), and the surface important for receptor binding is well exposed, but there is still not enough space for TMPRSS2 engagement.

Combining our current results with those of previous studies related to HKU1 receptor recognition^3^, we propose an updated model for dual receptor-based interaction between HKU1 and host cell with more details (Fig. 1n). HKU1 requires both glycan and proteinaceous receptors for spike-mediated cell attachment and entry. In the absence of glycan receptor, the HKU1 spike homotrimer adopts a closed conformation with all RBDs in the down position. Although the loop topologically equivalent to RBM (Insertion) is exceptionally large in HKU1, residues mediating TMPRSS2 binding are mainly gathered in a small groove (termed RBG) on the tip of RBD. RBG is not buried between adjacent S protomers in close state, but it cannot be engaged by TMPRSS2 due to steric hindrance. Binding of the glycan receptor (9-*O*-acetylated α2,8-linked sialic acid) to S1-NTD destabilizes close-state HKU1 spike and allosterically induces RBDs to flip up. Finally, RBD in the up position provides enough space for TMPRSS2 binding through interaction between HKU1 RBG and TMPRSS2 SP domains.

In summary, our findings offer a precise mapping of the HKU1–TMPRSS2 interaction, which is important for understanding the mechanism of HKU1 entry. Identification of key residues mediating spike–receptor binding provides a framework for vaccine and antiviral design. During the second revision of our manuscript, similar structures were also independently confirmed by three formally published articles^7,8,11^.

### Supplementary information

Supplementary Information

## Data Availability

The atomic models of HKU1A–TMPRSS2 and HKU1B–TMPRSS2 complex have been deposited into PDB with accession codes 8YOY and 8YQQ, respectively. The cryo-EM density maps have been deposited into the Electron Microscopy Data Bank under the accession codes EMD-39460 and EMD-39502, respectively.
