# Supplementary material for: Structural basis for the interaction between human coronavirus HKU1 spike receptor binding domain and its receptor TMPRSS2
Source: Cell Discov. 2024 Aug 8;10:84. doi: 10.1038/s41421-024-00717-5 (PMC11306395; doi:10.1038/s41421-024-00717-5)
Supplement: Supplementary file 1 — Supplementary Information [file 41421_2024_717_MOESM1_ESM.pdf]

Structural Basis for the Interaction between human coronavirus HKU1 Spike Receptor Binding Domain and its receptor TMPRSS2

Xiaopan Gao [1,2,5,#,*], Kaixiang Zhu [1,2,5,#], Lin Wang[4, 7,#] ,Kun Shang [3,4], Lei Hua[1,3], Bo Qin[1,2,5], Hongtao Zhu[4,6,7*] , Wei Ding[4,6,7*]and Sheng Cui [1,2,5,*]

[1]NHC Key Laboratory of Systems Biology of Pathogens, National Institute of Pathogen Biology, Chinese Academy of Medical Sciences and Peking Union Medical College, Beijing, China

[2]Key Laboratory of Pathogen Infection Prevention and Control (Peking Union Medical College), Ministry of Education, Beijing, China

[3]Medical School, Yan'an University, Yan'an, Shaanxi Province, China

[4] Beijing National Laboratory for Condensed Matter Physics, Institute of Physics, Chinese Academy of Sciences, Beijing, China

[5]State Key Laboratory of Respiratory Health and Multimorbidity, Chinese Academy of Medical Sciences and Peking Union Medical College, Beijing, China.

[6]University of Chinese Academy of Sciences, Beijing, China.

[7]Songshan Lake Materials Laboratory, Dongguan, Guangdong, China

[#]These authors contributed equally.

[*]Correspondence should be addressed to XG (gaoxiaopan@pumc.edu.cn),HZ (hongtao.zhu@iphy.ac.cn), DW (dingwei@iphy.ac.cn) and CS (cui.sheng@ipb.pumc.edu.cn)

Methods

Plasmid construction and protein purification

Coding sequences for human TMPRSS2 ectodomain (residues 109-492), HKU1 A RBD (residues 307-677) and HKU1 B RBD (residues 323-607) were synthesized by Genscript and subsequently cloned into the pFastBac1 vector (Supplementary Table S4, S5). The constructs included a hemo signal peptide at the N-terminus and a 6×His tag at the C-terminus. An S441A mutation was introduced into TMPRSS2 to inactivate the proteinase activity. The plasmids were then transformed into DH10Bac competent cells to generate recombinant bacmids. Recombinant bacmids were obtained through blue-white screening and then transfected into 0.8×10^6 sf-21 cells/9.6 cm^2 using FuGENE HD Transfection Reagent (Promega). After 90 hours of transfection, P1 viral stocks were collected and amplified to produce higher-titer P2 and P3 viral stocks. The P3 viral stock was added to High Five cells at a cell density of 2×10^6 cells/mL and cultured at 28°C with shaking at 120 rpm for 2 days.

For protein purification, cell cultures were centrifuged to remove cell debris and the supernatants were passed through Ni Sepharose excel (Cytiva) by gravity flow. Wash buffer (50 mM Tris-HCl, pH 8.0, 150 mM NaCl, 20 mM imidazole) was applied to remove non-specifically bound materials. The target proteins were eluted with elution buffer (50 mM Tris-HCl, pH 8.0, 50 mM NaCl, 300 mM imidazole). The samples were concentrated by ultrafiltration before loading onto a Superdex 200 Increase 10/300 GL column (Cytiva) pre-equilibrated in gel filtration buffer (20 mM

Tris-HCl, pH 8.0, 100 mM NaCl) for final purification. Peak fractions containing the target protein were concentrated and stored at -80°C for future use.

The molecular weight of purified TMPRSS2 ectodomain (residues 109-492), HKU1 A RBD (residues 307-677) and HKU1 B RBD (residues 323-607) estimated from size-exclusion chromatography is inconsistent with SDS-PAGE analyses (Supplementary Fig.S1 a-b). This might be stem from the shape of proteins and/or glycosylation during insect cells expression. To have more accurate measurement the molecular weights of these proteins, we carried out analytical ultracentrifugation (AUC). The results showed that the molecular weight of HKU1A-RBD, TMPRSS2 ectodomain and HKU1B-RBD of 49.4 kDa, 45.3 kDa, and 39.9 kDa (ordered by Mw from high to low), respectively; which is consistent with our SDS-PAGE analyses (Supplementary Fig.S1c).

To assemble the HKU1A RBD-TMPRSS2 and HKU1B RBD-TMPRSS2 complexes, the RBD was mixed with TMPRSS2 at a molar ratio of 2:1 (RBDs were in excess) and incubated at 4 °C for 6 h. The mixtures were then concentrated and loaded onto a Superdex 200 Increase 10/300 GL column pre-equilibrated in gel filtration buffer (20 mM Tris-HCl, pH 8.0, 100 mM NaCl) to separate the complexes from excess RBDs. Peak fractions containing the complexes were used for cryo-EM studies.

BLI assay

Biolayer interferometry (BLI) kinetic measurements were carried out using the ForteBio Octet RED96e Analysis System under standard conditions at 25°C. All

binding analyses were conducted in buffer A, which consisted of 20 mM HEPES at pH 7.4 and 100 mM NaCl. Purified TMPRSS2 was biotinylated using a biotinylation kit (Frdbio, ARL0020K) to enable immobilization on SA biosensors (Sartorius). Biotinylated TMPRSS2 at a concentration of 10 μg/mL was immobilized onto the SA biosensor for 200 seconds. Subsequently, the sensors were equilibrated in buffer A for an additional 200 seconds, and a baseline was recorded for 120 seconds before immersing them into various dilutions of HKU1A or HKU1B solutions ranging from 0 nM to 1000 nM. Following a 300-seconds association in the protein solution, dissociation was carried out in fresh buffer A for 600 seconds. The binding curves were analyzed using a 1:1 binding model with the ForteBio Data Analysis Software version 11.1, and all data were processed with reference well subtraction to ensure the accuracy of the results.

Cryo-EM Sample Preparation and Data Collection

The HKU1A RBD-TMPRSS2 and HKU1B RBD-TMPRSS2 complexes were concentrated to a final concentration of 0.3 to 0.5 mg/mL. Subsequently, 3 μL of each protein sample was immediately applied to glow-discharged Quantifoil R1.2/1.3 holey carbon Cu 300-mesh grids. After a 7-seconds waiting period, the grids underwent 4 blotting steps using a FEI Vitrobot under 100% humidity at 4°C. The grids were then plunge-frozen in liquid ethane, which had been pre-cooled by liquid nitrogen.

Cryo-EM data acquisition was performed at Shuimu BioSciences company. Specifically, images were collected on the FEI Titan Krios electron microscope (Thermo Fisher Scientific) operating at an acceleration voltage of 300 kV and equipped

with a Gatan K3 direct electron detector. Each movie stack was dose-fractionated into 32 frames with a total electron dose of 55 electrons/Å^2 and a total exposure time of 1.43 seconds, resulting in a physical pixel size of 0.81 Å/pixel. Data collection was carried out using EPU software with a defocus value ranging from -1.8 to -2.5 μm.

Cryo-EM Data Processing and 3D reconstruction

The data processing workflow involved the use of AutoEMage[1] and cryoSPARC v.4.4.1[2]. Dose-fractionated movies were collected and underwent patch motion correction in AutoEMage. Subsequently, the motion-corrected and dose-weighted micrographs were imported into cryoSPARC for determining the contrast transfer function (CTF) parameters through patch CTF estimation. Local resolution estimation was carried out using Local Resolution Estimation in cryoSPARC, followed by map sharpening with DeepEMhancer[3].

For the HKU1A-TMPRSS2 complex, a total of 6,799,626 particles were automatically picked from 8,999 micrographs and subjected to multiple rounds of 2D classification for cleaning. Subsequently, 2,034,986 selected particles underwent 3D Ab-Initio Reconstruction and Hetero refinement. A final round of 2D classification was used for particle selection, and 423,131 particles from the best class were utilized for 3D NU-refinement and Local refinement, resulting in a density map at a nominal resolution of 3.34 Å (determined by gold standard FSC using the 0.143 criterion; Fig. S2).

Regarding the HKU1B-TMPRSS2 complex, a total of 5,449,167 particles were automatically picked from 7,019 micrographs and cleaned through multiple rounds of

2D classification. Following this, 2,060,392 selected particles underwent 3D Ab-Initio Reconstruction and Heterogeneous refinement. Subsequently, one class was selected for further 2D classification, and particles from this class were used for 3D NU-refinement and Local refinement, resulting in a map at the resolutions of 3.95 Å for HKU1B-TMPRSS2(Supplementary Fig.S3).

Model Building and Refinement

Model building and refinement were conducted using the AlphaFold2 (AF2) predicted models of HKU1A, HKU1B and TMPRSS2 as initial models.[4] The HKU1B and TMPRSS2 models were firstly rigid body fitted into the density maps using UCSF Chimera[5] and adjusted manually using Coot[6]. Further refinement was carried out using phenix.real_space_refine [7]. Comprehensive model validation was performed using phenix.mtriage [7]. Despite the low resolution of the HKU1A RBD-TMPRSS2 density map, it is sufficiently resolved to allow unambiguous rigid body fitting of the HKU1A RBD and TMPRSS2 atomic model into the map position, obtaining the preliminary low-resolution model of HKUA1-TMPRSS2. The cryo-EM data collection, 3D reconstruction, and model refinement statistics are summarized in Supplementary Table 1. Structural figures were generated using PyMOL or ChimeraX[8].

Supplementary Figures

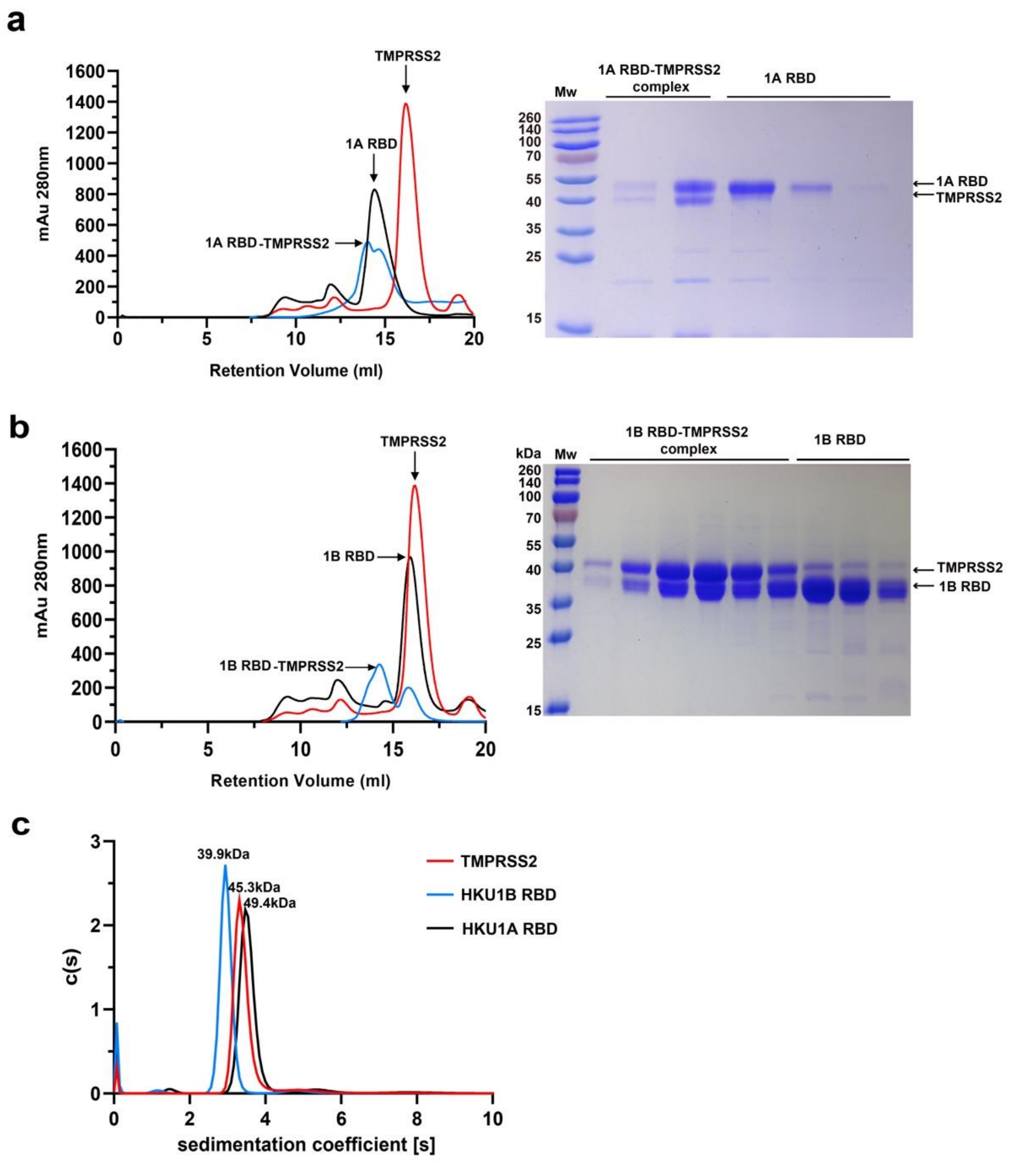

Figure S1. Assembly of HKU1 RBD-TMPRSS2 complexes.

a. In vitro assembly of HKU1A RBD (1A RBD)-TMPRSS2 complex.

b. In vitro assembly of HKU1B RBD (1B RBD)-TMPRSS2 complex.

c. Sedimentation analyses of HKU1A-RBD, TMPRSS2 (109-492) and HKU1B-RBD proteins used for complexes assembly; their calculated molecular weights are indicated, which are consistent with SDS-PAGE analyses (panel a and b).

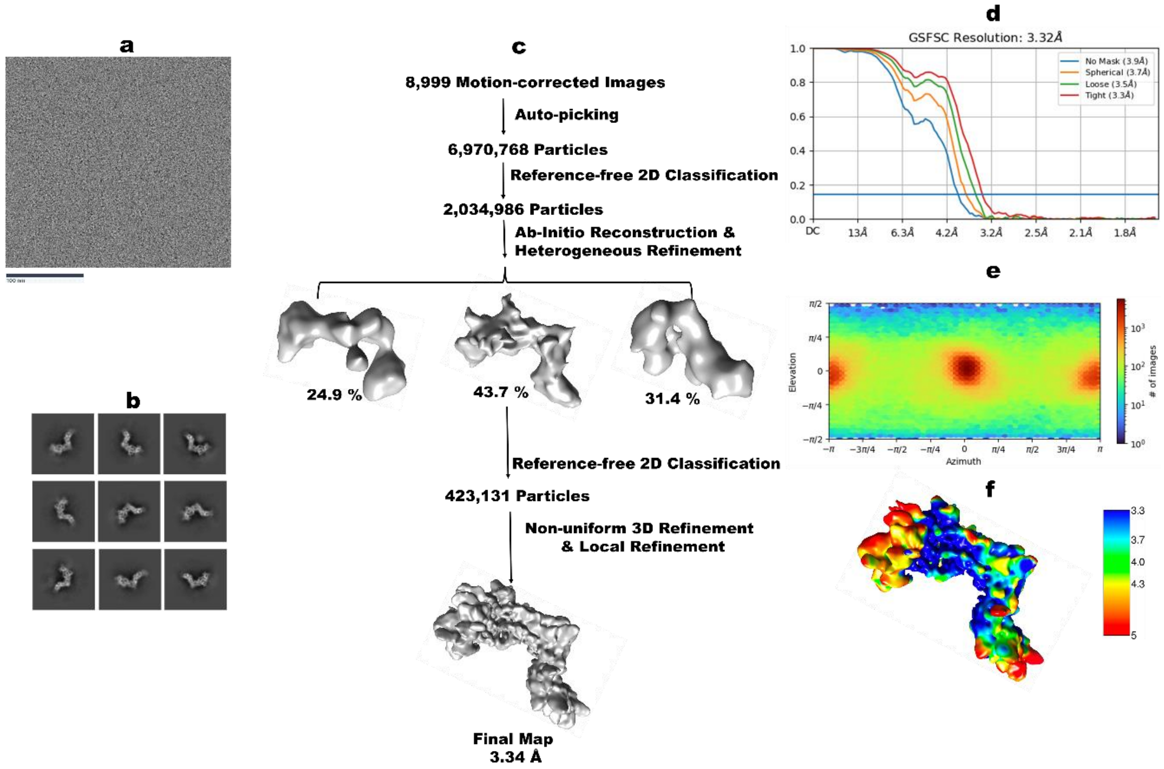

Figure S2 Cryo-EM data analysis workflow of HKU1A-TMPRSS2.

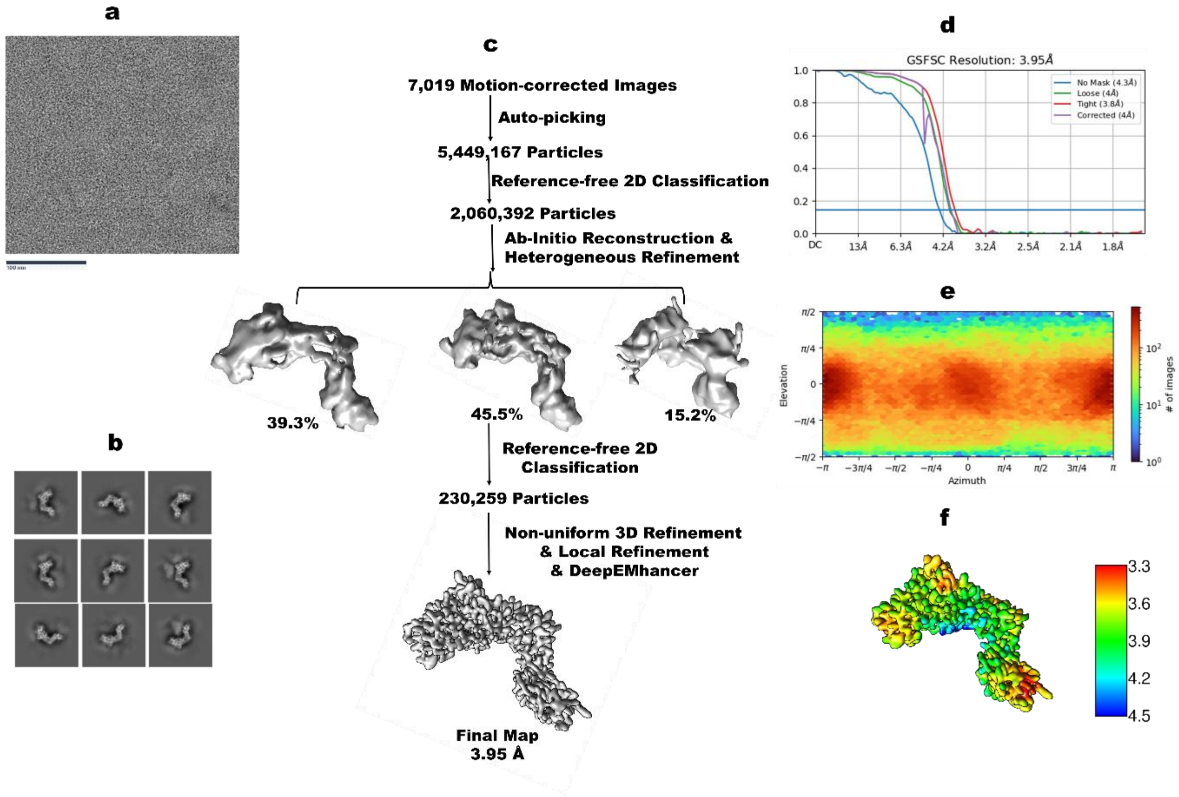

Figure S3 Cryo-EM data analysis workflow of HKU1B-TMPRSS2.

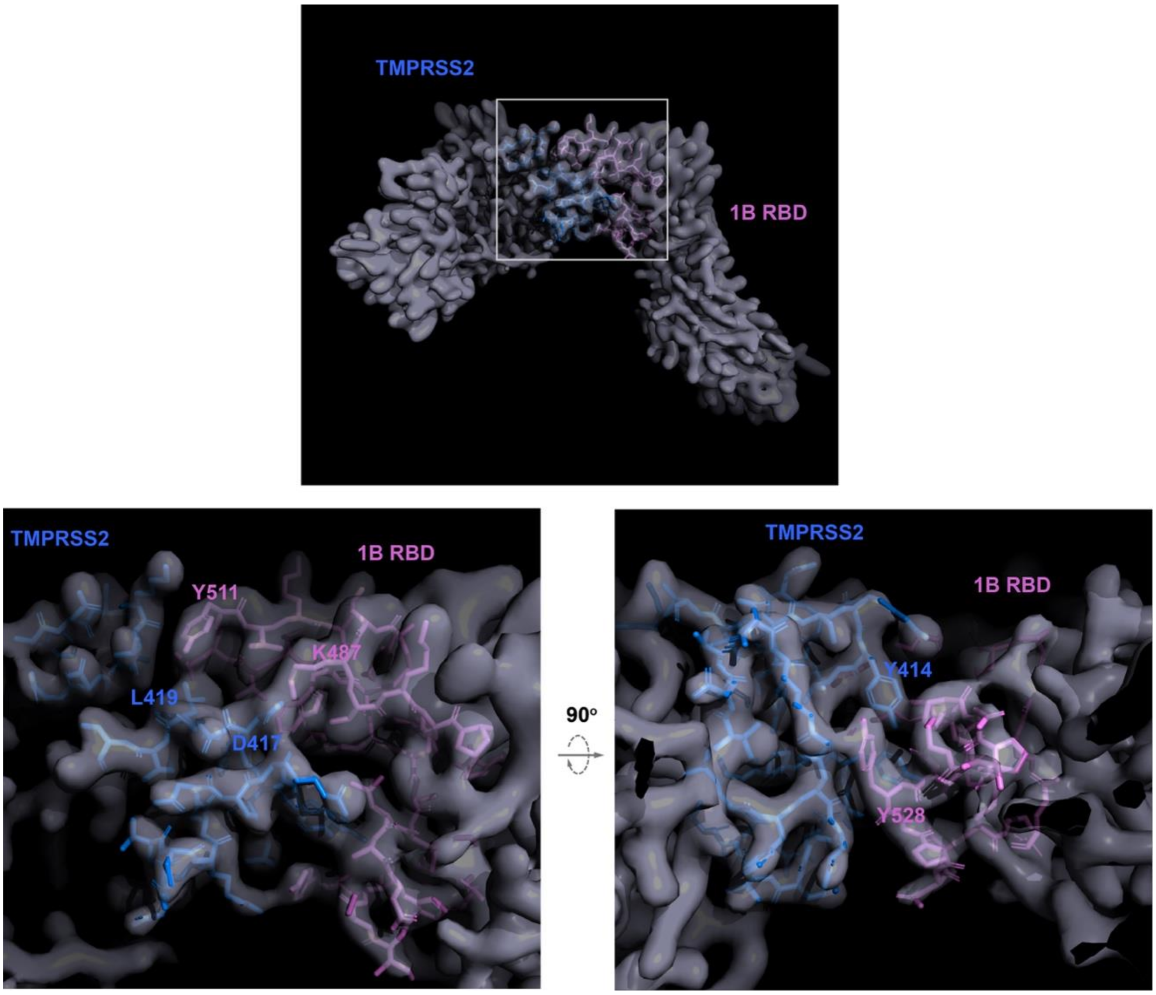

Figure S4 Cryo-EM density map of HUK1 1B RBD complexed with TMPRSS2

Cryo-EM density map (semitransparent surface) of TMPRSS2-1B RBD complex; residues at binding interface are shown with stick models (TMPRSS2, blue; 1B RBD, magenta).

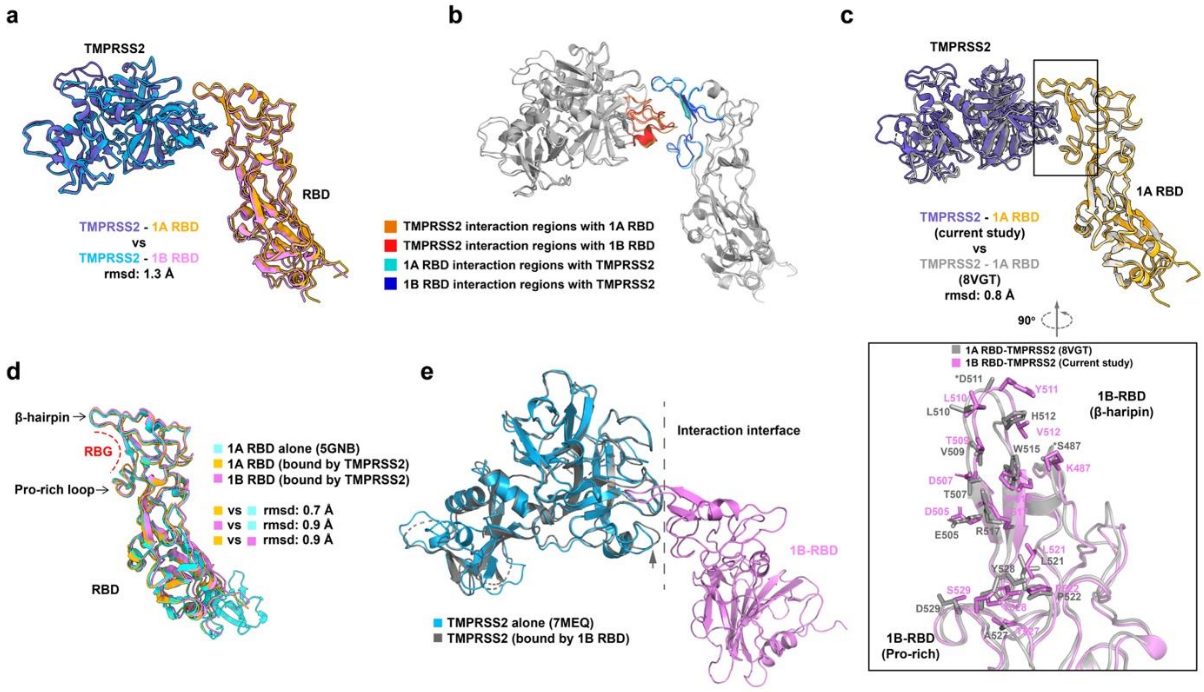

Figure S5. Superimposition of various HKU1 RBD TMPRSS2 structures

Structural comparison of various HKU1 RBD TMPRSS2 structures; RMSD values for structural superimposition are indicated.

a. Superimposition of 1A RBD-TMPRSS2 (determined in current study, purple-orange) with 1B RBD-TMPRSS2 (determined in current study, blue-magenta).

b. Structure of TMPRSS2 regions (residues 405-421, 427-436, 461-472, orange) directly interact with 1A RBD is superimposed with the structure of the same regions in TMPRSS2 that interacting with 1B RBD (red), which gave a RMSD value of 0.7Å. Structure of 1A RBD regions (residues 485-491, 502-535, cyan) directly interacting with TMRPSS2 is superimposed with the same region in 1B RBD (blue), which gave a RMSD value of 0.9Å.

c. Upper, superimposition of 1A RBD-TMPRSS2 (purple-orange) with a recently released 1A RBD-TMPRSS2 structure (gray, PDB, 8VGT). Lower, structural comparison of 1A (8VGT, gray) and 1B (current study, magenta) RBD with magnification at the receptor binding grooves (RBG) comprising a β-hairpin and a proline-rich region; key residues for receptor binding are shown with stick model and labeled. There are some incomplete model building in D511 and S487 of 8VGT, which are indicated by asterisks.

d. Comparison of free-state HKU1 S-RBD (PDB. 5GNB) with the receptor bound RBDs. RBG located on apex of RBD is indicated.

e. Comparison of free-state TMPRSS2 (gray, PDB. 7MEQ) with 1B RBD bound TMPRSS2 (blue-magenta). Interaction interface is indicated with a dashed line. The loop in TMPRSS2 undergoes evident conformational changes is indicated by an arrow.

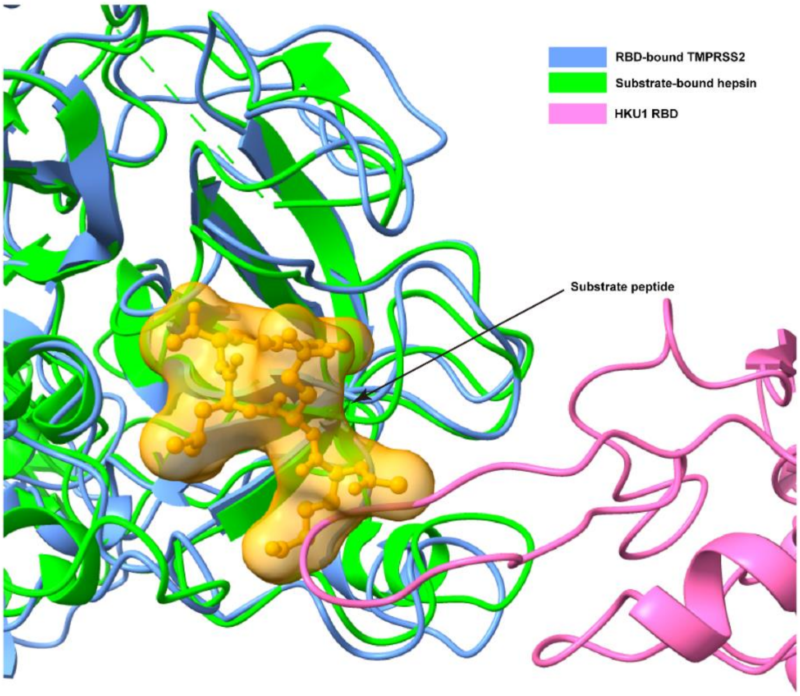

Figure S6. Superimposing the structure of hepsin bound to KQLR substrate (PDB ID 1Z8G) onto the structure of TMPRSS2 bound to HKU1 reveals steric hindrance that would prevent the ligand from binding in the active site upon attachment of the HKU1 RBD

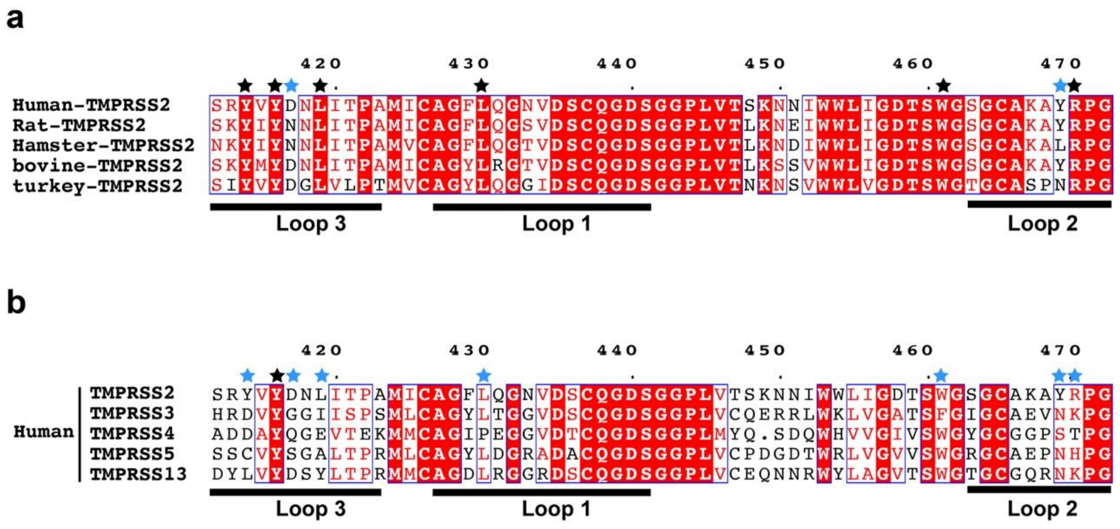

Figure S7. Analysis of HKU1 spike tropism for different TMPRSS2 and related proteins

Multiple sequence alignment of different TMPRSS2 and related proteins. Invariant residues are highlighted by red background, conserved residues are colored red. Stars indicate key residues (identified in our Cryo-EM structures) involved in binding HKU1 RBD; black stars indicate invariant residues involved in binding HKU1 RBD; blue stars indicate variable residues involved in binding. Three variable loops (Loop 1-3) of TMPRSS protein important to substrate recognition are in

a. Amino acid sequence alignment of TMRPSS2 and related protein from various species, including human, rat, hamster, bovine and turkey.
b. Amino acid sequence alignment of TMRPSS2-related proteins in human, including TMPRSS2, 3, 4, 5 and 13.

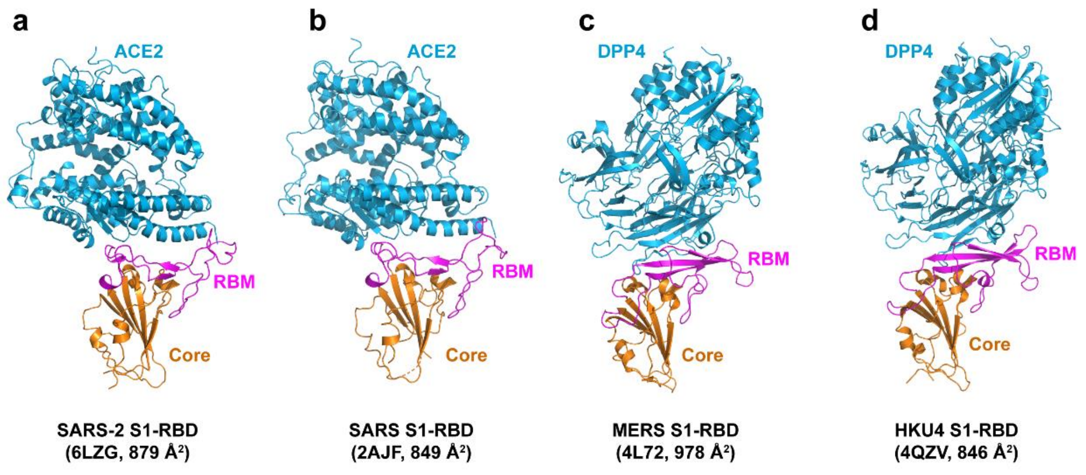

Figure S8. Structural comparison of various spike RBD-receptor complexes.

Structural comparison of various spike RBD-receptor complexes. RBD Core domain is colored orange, the receptor binding motifs (RBM) is colored magenta. The interface area between RBD and receptor are indicated.

a. SARS-CoV-2 S1-RBD-ACE2 complex (PDB, 6LZG);

b. SARS-CoV S1-RBD-ACE2 complex (PDB, 2AJF);

c. MERS-CoV S1-RBD-DPP4 complex (PDB, 4L72);

d. HKU4 S1-RBD-DPP4 complex (PDB, 4QZV).

Supplementary Table 1

Cryo-EM data collection parameters, refinement and validation statistics on reconstruction

Name	HKU1A-TMPRSS2	HKU1B-TMPRSS2
**PDB ID**	8YOY	8YQQ
**EMDB ID**	EMD-39460	EMD-39502
**Data collection and processing**		
Microscope	FEI Titan Krios	FEI Titan Krios
Voltage (keV)	300	300
Nominal Mag	96000x	96000x
Exposure navigation	Stage position	Stage position
Cumulative dose (e/Å^2)	55	55
Requested defocus range (um)	-1.8-2.5	-1.8-2.5
Detector	K3	K3
Pixel size (physical pixel, Å)	0.81	0.81
Total exposure time (sec)	1.43	1.43
Micrographs collected	8,999	7,019
**Reconstruction**		
Initial particles used	6,970,768	5,449,167
Particles selected after 2D classification	2,034,986	2,060,392
Particles used in final 3D reconstruction	423,131	230,259
Symmetry Imposed	C1	C1
Map Res (Å), masked/unmasked	3.3/3.9	3.95/4.3
FSC Threshold	0.143	0.143
Resolution range (local), Å	3.21-3.90	3.2-4.5
Final bfactor applied	-60	-60
**Model Refinement**		
Initial Model (PDB)	5GNB,7MEQ	AlphaFold2
Atoms	5081	5136
Protein residues	653	649
NAG	0	6
Map Correlation Coefficient	0.70	0.86
RMSD, Bond Lengths (Å)	0.005	0.04
RMSD, Bond Angles (°)	1.137	0.838
**Validation**		
Ramachandran Outliers (%)	0.00	0.00
Ramachandran Allowed (%)	4.31	4.50
Ramachandran Favored (%)	95.69	95.50
MolProbity score	1.72	1.67
Clashscore (all atoms)	7.59	6.40

Rotamer outliers (%)	0.00	0.00

Supplementary Table 2

Comparison of various HKU1 RBD-TMPRSS2 structures

**Structural superimposition between:**		**RMSD:**
1A-RBD-TMPRSS2<br>(Current study)	1B-RBD-TMPRSS2<br>(Current study)	1.3 Å
1A-RBD-TMPRSS2<br>(Current study)	1A-RBD-TMPRSS2<br>(PDB, 8VGT)	0.8 Å
1B-RBD-TMPRSS2<br>(Current study)	1A-RBD-TMPRSS2<br>(PDB, 8VGT)	1.1 Å
1A-RBD interaction regions	1B-RBD interaction region	0.9 Å
TMPRSS2 interaction region (bound by 1A RBD)	TMPRSS2 interaction region (bound by 1B RBD)	0.7 Å
TMPRSS2-1A RBD-complex (only the interaction regions of both parties)	TMPRSS2-1B RBD complex (only the interaction regions of both parties)	0.8 Å
1A RBD (bound by TMSRSS2, current study)	1A RBD alone (PDB, 5GNB)	0.7 Å
1B RBD (bound by TMSRSS2, current study)	1A RBD alone (PDB, 5GNB)	0.9 Å
1A RBD (bound by TMSRSS2, current study)	1B RBD (bound by TMPRSS2, current study)	0.9 Å
TMPRSS2 (bound by 1B RBD, current study)	TMPRSS2 alone (PDB, 7MEQ)	1.0 Å

Supplementary Table 3 Genetic polymorphisms of TMPRSS2 that cause missense mutation of the protein

**TMPRSS2 SNP (dbSNP number)**	**Missense mutation**	**References**
rs12329760	V197M; V160M	PMID: 36532428
rs75603675	G8V	PMID: 32691890
rs769655195	R255S	PMC7904510
rs1292701415	S441G	PMC7904510
rs61735791	A65T; A28T	PMC7904510

Supplementary Table 4

Synthesized genes in this study

Gene names	genes sequence
HKU1A-RBD	>TCAGGGTTCACAGTTAAGCCTGTGGCTACGGTGCATAGGAGGATTCCCGA<br>TCTGCCCGACTGCGATATCGACAAGTGGCTGAACAACTTCAACGTACCCTC<br>ACCGCTGAATTGGGAGAGGAAAATATTCTCCAATTGCAATTTTAATCTCTCC<br>ACTTTGCTGAGGCTGGTCCATACCGATTCATTTAGCTGTAATAACTTTGACG<br>AGAGTAAGATATATGGCAGCTGCTTCAAGAGCATTGTCCTGGACAAATTTG<br>CCATCCCTAACTCCAGGCGCTCAGATCTTCAACTGGGGAGCAGCGGATTCT<br>TGCAGAGCTCAAATTATAAAATCGATACCACAAGTAGCAGTTGTCAATTGTA<br>TTACTCACTGCCCGCTATTAACGTGACAATTAACAACTATAATCCTTCTTCTT<br>GGAACCGGCGGTACGGCTTCAACAACTTTAATTTGAGTAGCCACTCTGTGG<br>TGTACAGCAGATATTGTTTCTCAGTGAACAACACATTCTGCCCATGTGCCAA<br>GCCCAGCTTCGCAAGTAGCTGCAAGAGCCACAAGCCGCCATCCGCTTCTTG<br>TCCAATTGGGACCAACTACCGCTCTTGCGAAAGCACAACGGTGCTGGACC<br>ACACTGACTGGTGCAGATGTTCATGTCTGCCTGACCCAATTACCGCCTACG<br>ATCCCAGATCTTGCAGCCAGAAGAAATCTCTGGTCGGGGTCGGTGAACACT<br>GCGCGGGCTTTGGCGTGGATGAGGAAAAGTGCGGAGTCTTGGATGGGAGT<br>TACAATGTTAGTTGTTTGTGTAGTACTGATGCCTTCCTGGGATGGTCCTATGA<br>TACTTGTGTAAGTAACAATAGGTGCAATATTTTCAGTAACTTCATCTTGAAC<br>GGAATCAACAGTGGAACCACTTGTTCCAATGACCTGCTGCAACCTAATACG<br>GAAGTGTTTACCGACGTATGCGTAGATTACGACCTTTACGGCATAACAGGCC<br>AGGGGATCTTCAAGGAAGTATCCGCGGTGTACTATAACAGCTGGCAGAACC<br>TGCTGTACGACTCCAACGGCAACATCATTGGTTTCAAAGACTTCGTAACCA<br>ACAAGACATACAATATATTTCCATGCTACGCTGGT
HKU1B-RBD	>AATTTACCCGATTGCGACATAGATAATTGGTTGAATAACGTTTCAGTACCC<br>AGCCCTCTCAATTGGGAGCGACGTATTTTTTCAAATTGTAACTTTAACCTGT<br>CGACTCTCCTAAGGCTTGTCCACGTAGATTCGTTCTCTTGCAATAACCTTGA<br>CAAGTCCAAGATTTTTGGCAGTTGCTTCAACTCTATCACAGTGGATAAATTC<br>GCGATCCCTAACCGGAGAAGAGATGACCTACAGTTAGGCAGTTCCGGATTC<br>CTGCAATCATCAAACTATAAGATAGACATCAGTTCGAGCAGTTGTCAACTGT<br>ACTACAGCTTGCCCTTAGTTAATGTGACCATAAACAATTTCAACCCTTCTTC<br>GTGGAACCGGAGGTACGGTTTCGGTTCGTTTAACCTTTCCTCCTACGACGT<br>TGTCTATAGCGACCACTGCTTCAGCGTAAATTCTGATTTTTGTCCATGTGCT<br>GACCCGTCGGTTGTAAATTCATGCGCAAAAAGCAAACCGCCGTCCGCGATC<br>TGTCCTGCAGGGACAAAGTATCGCCATTGTGACCTGGATACTACCCTCTATG<br>TCAAAAATTGGTGCCGATGTTCTTGCTTGCCAGATCCAATTAGTACGTATAG<br>CCCTAACACGTGCCCGCAGAAAAAAGTGGTCGTGGGGATTGGCGAGCACT<br>GCCCAGGGCTAGGTATAAACGAAGAAAAGTGTGGTACACAATTGAATCATT<br>CTTCCTGCTTTTGTTCACCCGACGCCTTCCTTGGATGGTCATTTGACAGTTG<br>TATCTCGAATAACCGTTGCAATATTTTTAGTAATTTTATATTCAACGGAATAA<br>ATTCTGGCACGACCTGTTCCAACGATCTA

TMPRSS2(109-492)	>ATGGGCAGCAAGTGCTCCAACTCTGGGATAGAGTGCGACTCCTCAGGTACCTGCATCAACCCCTCTAACTGGTGTGATGGCGTGTCACACTGCCCCGGCGGGGAGGACGAGAATCGGTGTGTTCGCCTCTACGGACCAAACTTCATCCTTCAGGTGTACTCATCTCAGAGGAAGTCCTGGCACCCTGTGTGCCAAGACGACTGGAACGAGAACTACGGGCGGGCGGCCTGCAGGGACATGGGCTATAAGAATAATTTTTACTCTAGCCAAGGAATAGTGGATGACAGCGGATCCACCAGCTTTATGAAACTGAACACAAGTGCCGGCAATGTCGATATCTATAAAAAACTGTACCACAGTGATGCCTGTTCTTCAAAAGCAGTGGTTTCTTTACGCTGTATAGCCTGCGGGGTCAACTTGAACTCAAGCCGCCAGAGCAGGATTGTGGGCGGCGAGAGCGCGCTCCCGGGGGCCTGGCCCTGGCAGGTCAGCCTGCACGTCCAGAACGTCCACGTGTGCGGAGGCTCCATCATCACCCCCGAGTGGATCGTGACAGCCGCCCACTGCGTGGAAAAACCTCTTAACAATCCATGGCATTGGACGGCATTTGCGGGGATTTTGAGACAATCTTTCATGTTCTATGGAGCCGGATACCAAGTAGAAAAAGTGATTTCTCATCCAAATTATGACTCCAAGACCAAGAACAATGACATTGCGCTGATGAAGCTGCAGAAGCCTCTGACTTTCAACGACCTAGTGAAACCAGTGTGTCTGCCCAACCCAGGCATGATGCTGCAGCCAGAACAGCTCTGCTGGATTTCCGGGTGGGGGGCCACCGAGGAGAAAGGGAAGACCTCAGAAGTGCTGAACGCTGCCAAGGTGCTTCTCATTGAGACACAGAGATGCAACAGCAGATATGTCTATGACAACCTGATCACACCAGCCATGATCTGTGCCGGCTTCCTGCAGGGGAACGTCGATTCTTGCCAGGGTGACAGTGGAGGGCCTCTGGTCACTTCGAAGAACAATATCTGGTGGCTGATAGGGGATACAAGCTGGGGTTCTGGCTGTGCCAAAGCTTACAGACCAGGAGTGTACGGGAATGTGATGGTATTCACGGACTGGATTTATCGACAAATGAGGGCAGACGGC

Supplementary Table 5

List of primers used in this study.

Name	Forward primer (5'-3')	Reverse primer (5'-3')
pFastBac-hemo-HKU1A RBD-C-6His	GGTTCAGCGCTTCCGGGATCCTCAGGGTTCACAGTTAAGCCTG	CTTGGTACCGCATGCCTCGAGTTAGTGGTGGTGGTGGTGGTGACCAGCGTAGCATGGAAATATATTG
pFastBac-hemo-HKU1B RBD-C-6His	GGTTCAGCGCTTCCGGGATCCAATTTACCCGATTGCGACATAGA	CTTGGTACCGCATGCCTCGAGTTAGTGGTGGTGGTGGTGGTGTAGATCGTTGGAACAGGTCGTG
pFastBac-hemo-TMPRSS2-C-6His	GGTTCAGCGCTTCCGGGATCCATGGGCAGCAAGTGCTCCAAC	CTTGGTACCGCATGCCTCGAGTTAGTGGTGGTGGTGGTGGTGGCCGTCTGCCCTCATTTGTC
pFastBac-hemo-TMPRSS2(S441A)-C-6His	TTGCCAGGGTGACGCTGGAGGGCCTCTGGTCACTTC	CAGCGTCACCCTGGCAAGAATCGACGTTCCCC

References

1 Cheng, Y. H., Huang, X. J., Xu, B. & Ding, W. AutoEMage: automatic data transfer, preprocessing, real-time display and monitoring in cryo-EM. *Journal of Applied Crystallography* **56**, 1865-1873 (2023).

2 Punjani, A., Rubinstein, J. L., Fleet, D. J. & Brubaker, M. A. cryoSPARC: algorithms for rapid unsupervised cryo-EM structure determination. *Nat Methods* **14**, 290-296 (2017).

3 Sanchez-Garcia, R. *et al.* DeepEMhancer: a deep learning solution for cryo-EM volume post-processing. *Communications Biology* **4** (2021).

4 Jumper, J. *et al.* Highly accurate protein structure prediction with AlphaFold. *Nature* **596**, 583-589 (2021).

5 Pettersen, E. F. *et al.* UCSF Chimera--a visualization system for exploratory research and analysis. *J Comput Chem* **25**, 1605-1612 (2004).

6 Emsley, P., Lohkamp, B., Scott, W. G. & Cowtan, K. Features and development of Coot. *Acta Crystallogr D Biol Crystallogr* **66**, 486-501 (2010).

7 Adams, P. D. *et al.* PHENIX: a comprehensive Python-based system for macromolecular structure solution. *Acta Crystallogr D Biol Crystallogr* **66**, 213-221 (2010).

8 Pettersen, E. F. *et al.* UCSF ChimeraX: Structure visualization for researchers, educators, and developers. *Protein Sci* **30**, 70-82 (2021).
